# The Impact of Neighborhood Deprivation on the Survival Rates of Patients with Cancer in Korea

**DOI:** 10.3390/healthcare11243171

**Published:** 2023-12-15

**Authors:** Wonyoung Jung, Dong Wook Shin, Kyu-Won Jung, Dongjin Kim, Juwon Park, Fatima Nari, Mina Suh

**Affiliations:** 1 Department of Family Medicine/Obesity and Metabolic Health Center, Kangdong Sacred Heart Hospital, Hallym University, Seoul 05355, Republic of Korea; wyjung.md@gmail.com; 2 Department of Family Medicine and Supportive Care Center, Samsung Medical Center, Sungkyunkwan University School of Medicine, 81 Irwon-ro, Gangnam-gu, Seoul 06351, Republic of Korea; 3 Department of Clinical Research Design & Evaluation, Samsung Advanced Institute for Health Science & Technology (SAIHST), Sungkyunkwan University, Seoul 06355, Republic of Korea; 4 Korea Central Cancer Registry, National Cancer Center, Goyang 10408, Republic of Korea; ara@ncc.re.kr; 5 National Cancer Control Institute, National Cancer Center, 323 Ilsan-ro, Ilsandong-gu, Goyang 10408, Republic of Korea; jwpark7@ncc.re.kr (J.P.);; 6 Center for Health Policy Research, Korea Institute for Health and Social Affairs, Sejong 30147, Republic of Korea; djkim@kihasa.re.kr

**Keywords:** cancer, neighborhood deprivation index, social determinants of health, survival outcomes

## Abstract

The objective of this study is to investigate the correlation between the neighborhood deprivation index and survival rates of cancer patients in Korea. In this study, 5-year age-standardized survival rates of patients with cancer were determined using the National Cancer Cohort from 2014 to 2018 in Korea. The primary cancer sites were the stomach, colorectum, liver, lung, breast, cervix, prostate, and thyroid. Disparities were measured, and their impact on the overall survival rates was assessed using the Korean version of the Neighborhood Deprivation Index. Pearson’s correlation coefficient was calculated to determine the strength of the correlation. The study cohort comprised 726,665 patients with cancer, of whom 50.7% were male. The predominant primary cancer sites were the stomach (*n* = 138,462), colorectum (*n* = 125,156), and thyroid gland (*n* = 120,886). Urban residents showed better survival outcomes than those situated in rural areas. The most deprived quartile had the lowest survival rate, while the least deprived quartile had the highest (*p* < 0.001). Most cancer types revealed significant correlations between neighborhood deprivation and 5-year age-standardized overall survival, with lung cancer showing the most substantial negative correlation (r = −0.510), followed by prostate cancer (r = −0.438). However, thyroid cancer showed only a marginal correlation (*p* = 0.069). The results of this study suggested that neighborhood deprivation is closely linked to disparities in overall survival across various types of cancer. A substantial negative correlation between the neighborhood deprivation index and all-cause mortality for lung and prostate cancer, as compared to breast and cervical cancers covered by the National Cancer Screening Program, may reinforce the need to address healthcare access and improve the early detection of cancer in socioeconomically deprived neighborhoods.

## 1. Introduction

The 5-year relative survival rate of cancer patients in Korea has seen a significant and consistent increase, reaching 71.5% between 2016 and 2020, which has contributed to an increased prevalence rate of over 2.2 million in 2020 [1]. Yet, while cancer survival rates have shown improvements, ongoing challenges remain. In particular, socioeconomic disparities remain a significant concern, reflecting inequalities in the social determinants of health. Research has shown that neighborhood deprivation affects an individual’s overall health, often resulting in a vicious cycle of poor health, limited access to care, and continued poverty [2,3]. Specifically, cancer patients from underprivileged areas often experience various challenges, including lack of essential healthcare resources, fragmented social support systems, limited accessibility to medical facilities, and deprived resources [4,5]. This issue is further compounded by inequalities in education and employment [6]. All these factors can contribute to late-stage diagnoses and poor survival rates, primarily due to delayed screenings and inadequate early detection programs [7,8]. Moreover, these disparities persist even after considering individual-level factors such as insurance status, which suggests that neighborhood deprivation is an independent and significant determinant of health outcomes [9,10].

However, no study has yet investigated the association between neighborhood deprivation and cancer survival in Korea or assessed the differential associations by primary cancer site. Given the significant influence of socioeconomic factors on health outcomes, it is imperative to understand their specific effects on cancer survival in Korea. This study aims to identify such variables to facilitate appropriate intervention and the development of healthcare policies that reduce disparities in cancer outcomes.

In this context, this study aimed to investigate the correlation between the neighborhood deprivation index and cancer survival rates in Korea. Using nationwide data, this study assessed the associations among eight primary sites of cancer, i.e., the stomach, colorectal, liver, lung, breast, cervix, and prostate.

## 2. Materials and Methods

### 2.1. Data Source

This study used the National Cancer Cohort from 2014 to 2018. We linked data from the Korean Cancer Center Registry (KCCR) and the National Health Insurance Service (NHIS) claims databases to calculate the 5-year cancer survival rate in South Korea. The participants were followed-up until 31 December 2021. The KCCR data comprised the following relevant variables: sex; age at diagnosis; Surveillance, Epidemiology, and End Results (SEER) staging; and date of cancer diagnosis [11]. Moreover, the NHIS data comprised death records and various socioeconomic status (SES) variables of the subjects, such as income. The NHIS is a single insurer that provides medical insurance to approximately 97% of the population of Korea, in addition to managing the administration of medical aid to the population of patients with the lowest income levels. Therefore, the NHIS covers the entire Korean population. This dataset, which is rich in sociodemographic and diagnostic data, has been frequently used in epidemiological studies in Korea [12,13,14].

### 2.2. Study Population

This study enrolled patients diagnosed with cancer between 2014 to 2018 according to the diagnostic criteria of the International Classification of Diseases, 10th revision (ICD-10). Specific cancers included in the study were identified using the following codes: “C16” for stomach cancer, “C18–20” for colorectal cancer, “C22” for liver cancer, “C33–34” for lung cancer, “C50” for breast cancer, “C53” for cervical cancer, “C61” for prostate cancer, and “C73” for thyroid cancer.

### 2.3. Neighborhood Deprivation Index

In our study, we employed the Korean version of the Neighborhood Deprivation Index, developed by the Korea Institute for Health and Social Affairs [15]. This index is designed to quantitatively evaluate regional health disparities across Korea, offering a nuanced perspective on the effects of socioeconomic factors on health outcomes.

The index assesses seven domains, each concerned with a distinct aspect of social and economic life. The items in these domains amount to a total of 32 indicators. The domains and their corresponding indicators are as follows:

1. Economic (Income/Consumption): This domain’s indicators are income and consumption dissatisfaction, reflecting the economic well-being of a region and the confidence of the consumers who occupy it.
2. Employment: Employment indicators are the rate of non-participation in economic activity, the unemployment rate, the underemployment rate, the proportion of the population of working-age with an education level below college graduation, the rate of non-standard employment, and the rate of female non-participation in the labor force.
3. Education, Skills, and Occupational Training: The skill and education indicators are the proportion of students below a basic level of educational proficiency and the proportion of the population with a low level of education (below high school graduate level).
4. Health and Disability: The key health indicators are age-standardized mortality rates for various diseases, infant mortality rate, suicide mortality rate, poor self-perceived health rate, and depression rate.
5. Crime: The crime indicator is the number of crimes per thousand people.
6. Housing and Services: The indicators in this domain are the rate of non-enrollment in social insurance, the proportion of rental units in total housing stock, and the rate of unmet medical service needs.
7. Other Social Deprivation: The indicators of other social deprivation are the proportion of single-person households, the proportion of female-headed households, the divorce and bereavement rates, the proportion of elderly residents living alone, the traffic accident rate per thousand cars, the child accident mortality rate, the rate of non-receipt essential vaccinations, the proportion of residents receiving National Basic Support, and the non-voting rate.

The deprivation index is structured so that higher values could indicate increased levels of deprivation, with the scale ranging from −20.3312387 to 15.1452312. Korea comprises 250 municipal-level units (otherwise known as Si/Gun/Gu), and data on deprivation were provided for all municipal-level areas.

### 2.4. Cancer Survival Adjudication

The dependent variable was the 5-year age-standardized cancer survival rate, which was provided for all 250 municipalities in Korea. The 5-year cancer survival rate indicated the percentage of cancer patients alive five years after cancer diagnosis. In this study, cancer survival rates were calculated using the all-cause death records. Age standardization for survival rates was conducted using the approach proposed by Corazziari et al. through the incorporation of the International Cancer Survival Standard weights [16]. Moreover, the complete approach was used to estimate the 5-year survival for cancers that were diagnosed more recently (2014–2018), as this approach allows for the prediction of survival when 5 years of follow-up are not yet available [17].

### 2.5. Analytical Approach and Statistical Method

In this study, frequency and correlation analyses were performed. The relationship between the baseline characteristics of the study population and the 5-year overall survival rate was assessed using the chi-square test. For the analysis, the deprivation index was divided into quartiles (with the most deprived in Q1 to the least deprived in Q4). The Pearson correlation coefficient was calculated to determine the correlation between deprivation in the quartiles and the 5-year age-standardized cancer survival rate for each cancer type. Additionally, a correlation plot was drawn for the continuous values of deprivation indices and overall survival rates. All analyses were performed using the SAS statistical software package (version 9.4; SAS Institute, Cary, NC, USA).

### 2.6. Ethics Statement

This study used de-identified, publicly available data, rendering it exempt from the Institutional Review Board approval (approval number: NCC2021-0264, approval date: 24 August 2022).

## 3. Results

### 3.1. General Characteristics of Study Participants

In this study, the population comprised 726,665 cancer patients in Korea. The primary sites of cancer were the stomach (*n* = 138,462), colorectal (*n* = 125,156), thyroid (*n* = 120,886), lung (*n* = 109,116), breast (*n* = 99,553), liver (*n* = 70,712), prostate (*n* = 46,200), and cervix (*n* = 16,580) (Table 1).

The 5-year overall survival rate tended to be higher in males among survivors of stomach, colorectum, and liver cancers, whereas the opposite was observed among survivors of lung, breast, and thyroid cancers. Cancer survivors in older age groups were more likely to have lower 5-year overall survival rates across all seven primary cancer sites, with the exception of younger survivors (aged 20 years or less) of stomach and prostate cancers. Living in rural areas was associated with lower survival rates than those living in metropolitan or urban areas. The participants in the lowest quartiles of the deprivation index (most deprived) showed lower survival rates, whereas those in the highest quartiles (least deprived) had the highest survival rates (*p* < 0.001).

### 3.2. Correlation between Neighborhood Deprivation and 5-Year Age-Standardized Cancer Survival Rate

**Figure 1:**
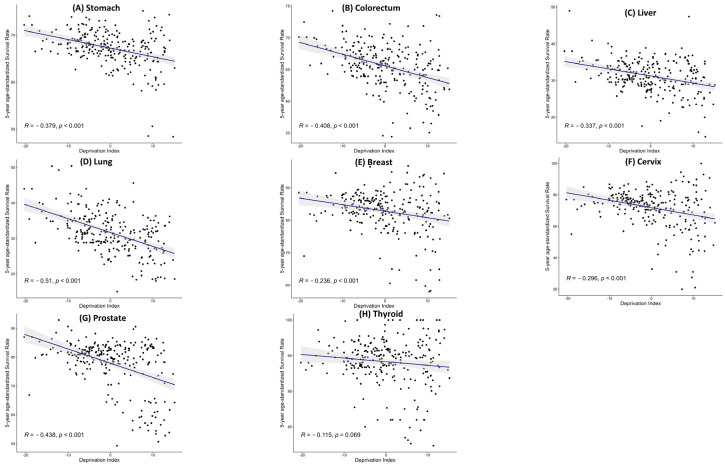
Correlation between the neighborhood deprivation index and site-specific 5-year cancer survival rate. (**A**) Stomach, (**B**) colorectum, (**C**) liver, (**D**) lung, (**E**) breast, (**F**) cervix, (**G**) prostate, and (**H**) thyroid. The points in the scatter plot show the relationship between the neighborhood deprivation index and 5-year age-standardized survival rate of 250 municipal-level units (Si/Gun/Gu). The lines indicate a linear regression line to demonstrate linearity and correlation.

Significant negative correlations between the neighborhood deprivation levels (measured in quartiles) and 5-year age-standardized cancer survival rates were observed for all specified cancer types (*p* < 0.001, Figure 1). Specifically, patients diagnosed with stomach, colorectal, liver, lung, breast, cervix, and prostate cancers showed statistically significant associations, wherein higher deprivation levels corresponded with lower survival rates. Lung cancer exhibited the highest negative correlation with deprivation (r = −0.510), followed closely by prostate cancer (r = −0.438), and colorectal cancer (r = −0.408). Stomach and liver cancers revealed moderate negative correlations (r = −0.379 and r = −0.337, respectively). Moreover, breast cancer and cervical cancer showed lower negative correlations (r = −0.230 and r = −0.296, respectively). By contrast, the negative correlation with thyroid cancer was insignificant (*p* = 0.069) (r = −0.115).

## 4. Discussion

In this cohort study of 726,665 cancer patients, it was observed that neighborhood socioeconomic deprivation negatively influenced the overall survival rates among multiple cancer types (stomach, colorectal, lung, breast, liver, prostate, and cervix cancers). Those in the most deprived areas showed the lowest survival rates among these cancer types, whereas their least deprived counterparts showed the highest survival rates. These findings are drawn from the large sample size of data from the KCCR and NHIS, providing sufficient statistical power to establish robust correlations.

The findings of this study confirm the growing body of evidence on the significant impact of neighborhood deprivation on cancer survival outcomes [2,18,19,20,21,22,23,24,25]. One of the most consequential impacts of neighborhood deprivation is the tendency toward late-stage cancer diagnosis. Residents in these areas often face barriers to early detection programs, such as inadequate screening initiatives and a lack of awareness of cancer warning signs [7,8]. Thus, this delay in diagnosis compromises prognosis and treatment options. Moreover, accessibility to comprehensive cancer care is significantly constrained in socioeconomically disadvantaged areas [26]. Factors such as geographical distance to healthcare facilities, financial limitations, and the scarcity of local healthcare resources limit the accessibility of necessary treatments [27,28]. Despite advancements in cancer treatments, including the use of novel chemotherapeutic agents and immunotherapy, the associated costs can be prohibitive, although certain programs have been implemented to alleviate the financial burden of cancer patients [29,30,31,32]. Interestingly, in Korea, the issue extends beyond finances, considering that cancer patients are only responsible for 5% of their cancer-related medical bills with an applicable co-payment reduction code (V193). This suggests the presence of other socioeconomic barriers that impede healthcare access. Meanwhile, the socioeconomic makeup of deprived neighborhoods often correlates with unhealthy lifestyles, such as poor diet, physical inactivity, and tobacco use, which are known risk factors for various cancers [27,28]. These communities also frequently contend with environmental hazards, including higher levels of pollution, which may independently increase the cancer risk [33]. However, it is important to note that our outcomes were overall outcomes, which did not detect cancer-specific mortality.

Our analysis revealed distinct degrees of correlation across the different cancer types. Lung cancer was significantly negatively correlated (r = −0.510) with the neighborhood deprivation index, suggesting a critical influence on survival outcomes. In South Korea, the absence of the National Cancer Screening Program (NCSP) before 2019 implies that the early detection of cancer is often facilitated through private screenings, which are more frequently utilized in affluent urban areas. Given that lung cancer is typically characterized by poor prognosis, largely due to late-stage diagnosis, the disparity in early detection is particularly notable. Similarly, prostate cancer, which is often detected during private screenings and is associated with a better prognosis when diagnosed early, also showed a significant negative correlation (r = −0.438). This pattern emphasizes the importance of early detection, particularly in cancers in which effective treatment is highly dependent on the stage at diagnosis. Notably, prostate cancer screening is not included in the NCSP; therefore, early detection and access to healthcare facilities are more likely to impact prognosis.

Conversely, cancers such as breast and cervical cancers, which benefit from established national screening programs, showed weaker negative correlations (r = −0.230 and r = −0.296, respectively). These findings suggest that organized screening programs enhance early detection across socioeconomic boundaries, subsequently influencing survival rates by allowing for timely intervention. However, despite its inclusion in Korea’s NCSP, colorectal cancer showed a negative correlation with the Neighborhood Deprivation Index (r = −0.408), similar to that of prostate cancer. This similarity implies potential underlying factors affecting survival outcomes beyond the availability of early screening. In South Korea, among the five major cancers (stomach, colorectum, lung, breast, and cervix) included in the NCSP, colorectal cancer screening has the lowest participation rate, which varies with the income level [34]. In the NSCP, implementation of the fecal immunochemical test has been encouraged since 2004 as a primary screening method for colorectal cancer. This program was anticipated to level the field of early cancer detection across different socioeconomic groups. Despite this, people with lower income brackets participate in colorectal cancer screenings only at a significantly reduced rate compared to their higher-income counterparts (51.7% vs. 62.0%) [35]. This gap in screening participation emphasizes persistent health inequality. Even with a national program in place, factors such as the inadequate understanding of screening benefits, financial problems, inaccessibility of healthcare facilities, and inconsistent trust in healthcare providers discourage those with lower incomes from being screened [35,36]. This may result in differences in the survival rates of patients with colorectal cancer across socioeconomic groups.

Interestingly, thyroid cancer showed the weakest negative correlation (r = −0.115). This weak association could be related to the generally favorable prognosis of thyroid cancer, with high survival rates irrespective of SES, coupled with less reliance on the early detection for successful treatment outcomes. By contrast, liver and stomach cancers showed moderate negative correlations (r = −0.337 and r = −0.379, respectively). This finding suggests that, unlike thyroid cancer, the disparities in the survival rates for these cancers may be influenced by certain factors, such as NSCP or healthcare accessibility, thereby affecting the observed correlation.

Our findings also have clinical implications for the health disparities in cancer outcomes. Notably, Korean legislation has proactively recognized disparities in cancer outcomes by initiating nationwide screening programs and claim-based billing to alleviate the financial burden on cancer patients. To increase the financial protection available to cancer patients, the Korean government has already expanded the benefit coverage of national health insurance and reduced the out-of-pocket healthcare expenses of cancer patients for 5 years from the date of registration [37]. However, even with such initiatives, a disparity in survival rates can be observed, particularly among deprived individuals. Our findings suggest that broader socioeconomic determinants, such as education, employment, and access to social support services, influence cancer survival.

To address these disparities, a balanced approach involving both governmental and non-governmental organizations (NGOs) is necessary. Governmental strategies and policies aimed at increasing equitable healthcare access and providing free cancer screening programs in socioeconomically disadvantaged areas are needed. These should be complemented via NGO provision of community support such as educational campaigns and advocacy for improved healthcare services in underserved communities. The coordination of screening programs with local community resources could also help bridge the gap in healthcare access. Cooperative synergy between such government and NGO initiatives can vastly reduce current healthcare disparities [38,39]. The integration of these efforts is expected to gradually improve cancer survival rates across socioeconomic groups. 

However, this study had several limitations. First, our analysis was primarily based on data from Korea, making it potentially less applicable to the context of other cultural or healthcare systems. Second, our correlation analysis at the area level did not adjust for potential confounders known to affect cancer survival, such as mutational burden, regional variations in lifestyle habits (e.g., smoking, alcohol consumption, and diet) and levels of environmental pollution. Third, this study did not address the potential variations in cancer subtypes and stages at diagnosis among different socioeconomic groups, which could have contributed to the differences in survival outcomes. Lastly, the survival statistics used in our analysis were derived from all-cause death records rather than from cancer-specific survival data.

## 5. Conclusions

This study confirms the influence of neighborhood socioeconomic deprivation on cancer survival rates, even in the context of a nation with proactive healthcare initiatives, such as Korea. Cancer patients from the most deprived neighborhoods had the lowest cancer survival rates compared with those from the least deprived neighborhoods. Moreover, our results suggest that neighborhood deprivation is closely linked to disparities in overall survival across various types of cancers. A substantial negative correlation between the neighborhood deprivation index and all-cause mortality for lung and prostate cancers, compared with breast and cervical cancers, which are covered by the NCSP, may reinforce the need to address healthcare access and improve the early detection of cancer especially in socioeconomically deprived neighborhoods.

## Figures and Tables

**Table 1:** General characteristics of participants and 5-year age-standardized overall survival rates by cancer type (2014–2018).

	Stomach (*n* = 138,462)		Colorectum (*n* = 125,156)		Liver (*n* = 70,712)		Lung (*n* = 109,116)		Breast (*n* = 99,553)		Cervix (*n* = 16,580)		Prostate (*n* = 46,200)		Thyroid (*n* = 120,886)	
	N (%)	5-OS (SD)	N (%)	5-OS (SD)	N (%)	5-OS (SD)	N (%)	5-OS (SD)	N (%)	5-OS (SD)	N (%)	5-OS (SD)	N (%)	5-OS (SD)	N (%)	5-OS (SD)
Sex																
Male	93,240 (67.34)	70.44 (0.15)	74,086 (59.19)	67.79 (0.18)	52,846 (74.73)	34.82 (0.22)	75,225 (68.94)	24.61 (0.16)	394 (0.40)	80.80 (2.08)	-	-	46,200 (100.0)	81.44 (0.19)	26,246 (21.71)	98.04 (0.09)
Female	45,222 (32.66)	69.97 (0.22)	51,070 (40.81)	66.45 (0.22)	17,866 (25.27)	32.61 (0.36)	33,891 (31.06)	43.18 (0.28)	99,159 (99.60)	91.82 (0.09)	16,580 (100.0)	78.23 (0.33)	-	-	94,640 (78.29)	98.83 (0.04)
Age																
≤20	377 (0.27)	61.38 (2.53)	614 (0.49)	81.26 (1.51)	126 (0.18)	49.12 (4.64)	190 (0.17)	67.81 (3.45)	853 (0.86)	90.68 (1.04)	464 (2.80)	85.71 (1.66)	11 (0.02)	18.18 (11.63)	9271 (7.67)	99.85 (0.05)
30	3474 (2.51)	75.04 (0.75)	3120 (2.49)	83.81 (0.68)	1298 (1.84)	42.31 (1.40)	871 (0.80)	54.13 (1.76)	9280 (9.32)	92.56 (0.29)	2879 (17.36)	88.79 (0.61)	15 (0.03)	80.00 (10.33)	24,884 (20.58)	99.83 (0.03)
40	13,769 (9.94)	81.39 (0.34)	10,642 (8.50)	80.79 (0.40)	7247 (10.25)	43.44 (0.60)	4297 (3.94)	53.25 (0.80)	33,737 (33.89)	94.91 (0.13)	4346 (26.21)	85.43 (0.55)	363 (0.79)	89.80 (1.62)	34,777 (28.77)	99.66 (0.03)
50	32,143 (23.21)	80.45 (0.23)	27,669 (22.11)	79.99 (0.25)	19,207 (27.16)	43.20 (0.37)	16,388 (15.02)	48.49 (0.41)	30,262 (30.40)	92.96 (0.16)	3863 (23.30)	82.10 (0.64)	4898 (10.60)	92.10 (0.41)	31,819 (26.32)	99.19 (0.05)
60	38,230 (27.61)	79.24 (0.21)	32,491 (25.96)	76.08 (0.25)	18,662 (26.39)	40.36 (0.37)	30,510 (27.96)	39.08 (0.29)	15,909 (15.98)	91.99 (0.23)	2257 (13.61)	79.63 (0.87)	16,168 (35.00)	90.96 (0.24)	14,666 (12.13)	97.89 (0.13)
≥70	50,469 (36.45)	53.71 (0.23)	50,620 (40.45)	50.54 (0.23)	24,172 (34.18)	19.04 (0.27)	56,860 (52.11)	18.22 (0.17)	9512 (9.55)	75.77 (0.47)	2771 (16.71)	48.24 (0.98)	24,745 (53.56)	72.96 (0.31)	5469 (4.52)	83.85 (0.52)
Region																
Metropolitan	58,382 (42.16)	71.62 (0.19)	54,786 (43.77)	68.83 (0.21)	29,983 (42.40)	34.90 (0.29)	45,451 (41.65)	33.04 (0.23)	47,424 (47.64)	92.11 (0.13)	7378 (44.50)	80.26 (0.47)	20,487 (44.34)	82.69 (0.28)	58,211 (48.15)	98.74 (0.05)
Rural	19,335 (13.96)	63.87 (0.35)	15,945 (12.74)	60.84 (0.40)	10,285 (14.54)	30.58 (0.47)	17,004 (15.58)	23.13 (0.34)	7211 (7.24)	88.64 (0.39)	1558 (9.40)	70.53 (1.19)	6165 (13.34)	76.92 (0.57)	8898 (7.36)	97.61 (0.17)
Urban	60,745 (43.87)	71.05 (0.19)	54,425 (43.49)	67.52 (0.21)	30,444 (43.05)	34.87 (0.28)	46,661 (42.76)	30.42 (0.22)	44,918 (45.12)	91.93 (0.14)	7644 (46.10)	77.85 (0.49)	19,548 (42.31)	81.56 (0.30)	53,777 (44.49)	98.75 (0.05)
Deprivation																
Q1	14,948 (10.80)	63.44 (0.40)	11,985 (9.58)	59.92 (0.46)	7975 (11.28)	29.64 (0.53)	13,107 (12.01)	22.32 (0.38)	4849 (4.87)	88.21 (0.49)	1136 (6.85)	70.07 (1.39)	4634 (10.03)	75.42 (0.68)	6784 (5.61)	97.39 (0.20)
Q2	26,606 (19.22)	67.95 (0.29)	23,767 (18.99)	65.19 (0.32)	14,150 (20.01)	32.80 (0.41)	21,647 (19.84)	27.38 (0.32)	15,234 (15.30)	90.41 (0.25)	2904 (17.52)	75.29 (0.82)	9046 (19.58)	79.77 (0.45)	18,246 (15.09)	98.42 (0.10)
Q3	48,845 (35.28)	71.06 (0.21)	45,537 (36.38)	67.73 (0.23)	25,176 (35.60)	34.46 (0.31)	38,355 (35.15)	30.75 (0.25)	36,594 (36.76)	91.79 (0.15)	6223 (37.53)	77.63 (0.54)	15,925 (34.47)	82.00 (0.33)	44,265 (36.62)	98.69 (0.06)
Q4	48,063 (34.71)	72.94 (0.21)	43,867 (35.05)	69.85 (0.23)	23,411 (33.11)	36.50 (0.33)	36,007 (33.00)	34.70 (0.26)	42,876 (43.07)	92.66 (0.13)	6317 (38.10)	81.65 (0.50)	16,595 (35.92)	83.50 (0.31)	51,591 (42.68)	98.89 (0.05)

Q1, Q2, Q3, and Q4 represent the quartiles of neighborhood deprivation index used in our analysis. Q1: most deprived; Q4: least deprived; 5-OS, 5-year overall survival; SD, standard deviation. For all statistical analyses, *p* < 0.001.

## Data Availability

The data will be made available upon request and approval of a proposal by the Korea Central Cancer Registry and the National Health Insurance Service Database.
